# Supplementary material for: Strain-graded quantum dots with spectrally pure, stable and polarized emission
Source: Nat Commun. 2024 Jul 2;15:5561. doi: 10.1038/s41467-024-49791-z (PMC11220006; doi:10.1038/s41467-024-49791-z)
Supplement: Supplementary file 1 — Supplementary information [file 41467_2024_49791_MOESM1_ESM.pdf]

Supplementary Information

Strain-graded quantum dots with spectrally pure, stable and polarized emission

Dongju Jung[1†], Jeong Woo Park[1†], Sejong Min[1], Hak June Lee[1], Jin Su Park[1], Gui-Min Kim[2], Doyoon Shin[1], Seongbin Im[1], Jaemin Lim[1], Ka Hyung Kim[1], Jong Ah Chae[5], Doh C. Lee[2], Raphaël Pugin[3], Xavier Bulliard[3], Euyheon Hwang[1], Ji-Sang Park[1*], Young-Shin Park[4*], and Wan Ki Bae[1,5*]*

[1]SKKU Advanced Institute of Nanotechnology (SAINT), Sungkyunkwan University (SKKU), Suwon 16419, Republic of Korea

[2]Department of Chemical and Biomolecular Engineering, KAIST Institute for the Nanocentury, Korea Advanced Institute of Science and Technology (KAIST), Daejeon 34141, Republic of Korea

[3]Centre Suisse d'Electronique et de Microtechnique (CSEM SA), CH-2002 Neuchatel, Switzerland

[4]Chemistry Division, Los Alamos National Laboratory, Los Alamos, NM 87545, United States

[5]Department of Display Engineering, Sungkyunkwan University (SKKU), Suwon 16419, Republic of Korea

†These authors contributed equally to this work.

Correspondence to Wan Ki Bae (wkbae@skku.edu); Young-Shin Park (youngshin19@gmail.com); Ji-Sang Park (jisangpark@skku.edu); Xavier Bulliard (xavier.bulliard@csem.ch)

<u>Supplementary Note 1.</u> Calculation of surface energy

Density functional theory (DFT) calculations were performed to obtain surface energies of CdSe. We used an exchange-correlation functional parametrized by Perdew, Burke, and Erzernhof (PBE)[1] and projector-augmented wave (PAW) method[2], as implemented in the Vienna Ab-initio Simulation Package (VASP)[3]. The atomic structures were optimized until the residual force became less than 0.01 eV/Å. The cell parameters and the internal coordinates of bulk CdSe were optimized by expanding the plane waves up to 342.9 eV. Otherwise, the energy cutoff was set to 300 eV.

Atomic structure models of CdSe (0001) and CdSe ($000\bar{1}$) surfaces are shown below. 100 % terminated CdSe (0001) has undercoordinated Cd atoms, while 100 % terminated CdSe ($000\bar{1}$) surface has Se dangling bonds. For each model, we introduced a vacancy defect at the surface for every four undercoordinated atoms, making partially terminated models. In every model, one side of the slabs was fully passivated by pseudo-hydrogen atoms, and the thickness of the vacuum region was thicker than 15 Å.

Our calculation shows that vacancy formation reduces the surface energies. In a Cd-rich condition, in which the formation of Cd vacancies is least favorable, the 75 % Cd-terminated surface has 2.60 eV/nm^2 lower surface energy than the 100 % Cd-terminated surface. Similarly, in a Se-rich condition, the 75 % Se-terminated model has 1.19 eV/nm^2 lower surface energy than the 100 % Se-terminated model. We expect that the Se vacancies will be occupied by the supplied Se atoms during the subsequent ZnSe growth while Zn atoms are to be adsorbed on top of CdSe, making an abrupt CdSe/ZnSe interface.

<u>Supplementary Note 2.</u> Calculation of critical thickness of ZnSe shell layer on CdSe

The critical thickness for the ZnSe shell grown on the CdSe core is estimated by following equation (1) from Matthew and Blakeslee's equilibrium theory[4].

$$h_c = \frac{b}{2\pi f}\frac{(1-\nu\cos^2\alpha)}{(1+\nu)\cos\lambda}(\ln\frac{h_c}{b}+1) \quad (1)$$

where *b* is the magnitude of Burgers vector (b = 1/3 $<\bar{1}2\bar{1}0>$ for wurtzite (wz) crystal)[5], *f* is the lattice mismatch between the film and the substrate (-5.48 % along [0002] and -7.05 % along [1000], respectively)[6], ν is the Poisson ratio (0.31 for [0002] and 0.37 for [1000], respectively)[7] and α is the angle between the dislocation line and its Burgers vector (60 °)[5]. λ is the angle between the slip direction and the direction in the film plane which is perpendicular to the line of intersection of the slip plane and the interface (45 °)[4]. The calculated critical thicknesses of the ZnSe layer on the CdSe core are 2.33 nm along [0002] and 1.23 nm along [1000], respectively.

Supplementary Note 3. Asymmetric strain of CdSe/ZnSe interface

The biaxial compressive strain of CdSe by ZnSe shell was modeled by performing DFT calculations. In our CdSe/ZnSe interface models, the number of ZnSe layers is increased from 0 to 24, while the number of CdSe layers is kept to 12. Since the interface normal direction is along $[10\bar{1}0]$ (*A axis*), we observe the biaxial compressive strain along [0001] (*C axis*) and $[1\bar{2}10]$ direction, which is perpendicular to the *A* and *C* axes. The lattice contracts along both directions because of the smaller lattice constants of ZnSe. Initially, the strain along the $[1\bar{2}10]$ direction changes more rapidly compared to the *C* axis, creating strain asymmetry. However, the difference between these directional strains diminishes as the ZnSe layers are added. This computational finding is in good agreement with the observed increase and subsequent decrease in strain asymmetry, while the overall lattice is still more compressively strained.

<u>Supplementary Note 4.</u> Calculation of bulk bandgap of strained CdSe and volumetric strain

To examine the effect of volumetric strain on the band gap of bulk CdSe, we performed hybrid DFT calculations using the exchange-correlation functional parametrized by Heyd, Scuseria, and Ernzerhof (HSE)[8] and PAW method[2], as implemented in the VASP[3]. The cell parameters and the internal coordinates were optimized by applying hydrostatic pressure until the residual force became less than 0.01 eV/Å. The energy cutoff was set to 342.9 eV during the cell optimization. The electronic band gap was obtained by setting the energy cutoff to 300 eV. As shown in **Supplementary Table 1**, the calculated band gap monotonically increases as the compressive strain is applied to the lattice, in good agreement with the increased absorption peak in the experiments.

Supplementary Table 1 | The band gap of strained CdSe obtained by performing hybrid DFT calculations. The hydrostatic compressive strain was applied to the lattice. The band gap increases as the lattice constants a and c decrease.

$\Delta a/a$ (%)	$\Delta c/c$ (%)	Band gap (eV)
-4.8	-4.2	1.90
-3.7	-3.6	1.84
-2.7	-2.6	1.76
-1.5	-1.4	1.67
0	0	1.55

The bulk energy level of the strained CdSe was estimated by solving the Schrodinger's equation in the two bands k · p method. Conduction band minimum (CBM) and valence band maximum (VBM) of bulk CdSe were assumed to change linearly with the lattice deformation. From the shell etching experiment, we could conclude that photoluminescence (PL) shift upon the ZnSe shell growth comes from the lattice strain of the CdSe core. In a given geometry of CdSe-ZnSe QDs (*i.e.,* CdSe core radius (r = 2.0, 2.5, 3.0, 3.5 and 4.0 nm) and ZnSe shell thickness (H = 1.0, 2.0, 3.0, 4.0 and 5.0 nm)), we modified the bulk bandgap of CdSe core ($\Delta E_{g,Bulk}$) until the energy gaps between lowest quantized states for electron and heavy hole obtained from calculations agree with $1S_e$-$1S_{HH}$ energy gaps of CdSe-ZnSe QDs gained from the absorbance and photoluminescence spectra (**Fig. 2c** of the main text).

The effective compressive strain (β^*) were estimated from the changes in the bulk bandgap ($\Delta E_{g,Bulk}$) using the following equation (2)[9].

$$\Delta E_{g,bulk} = a_{deformation} * \varepsilon_{volumetric} \quad (2)$$

where $E_{g,Bulk}$ is the bulk bandgap of wurtzite CdSe, $a_{deformation}$ is deformation potential (-2.3 eV for wurtzite CdSe) and $\varepsilon_{volumetric}$ is the volumetric strain.

Supplementary Note 5. Calculation of phonon energy

Optical phonon modes of CdSe at the Γ point were obtained by performing DFT calculations. We used the PBE exchange-correlation functional[1] and PAW method[2], as implemented in the VASP[3]. The cell parameters and the internal coordinates were optimized by applying hydrostatic pressure until the residual force became less than 0.01 eV/Å. The energy cutoff was set to 342.9 eV during the cell optimization. We employed a 192-atom supercell to obtain converged vibrational modes. In the supercell calculations, plane waves were expanded to 300 eV, and a 3×3×3 *k*-point grid was used for the Brillouin zone integration. Phonopy was used to obtain the phonon band structure[10]. As summarized in **Supplementary Table 2**, the optical phonons at the Γ point were hardened by 2 and 6 meV on average when the compressive strain was applied by 1.6 % and 5 %, respectively.

Supplementary Table 2 | Calculated longitudinal optical (LO) and transverse optical (TO) phonon energy and with respect to lattice strain.

Lattice strain (%)	Phonon energy (meV)					
	LO	TO	TO	TO	TO	LO
0	19.09	19.71	19.71	19.99	19.99	21.7
-1.6	21.01	21.6	21.6	22.09	22.09	24.03
-5	25.27	25.64	25.64	26.56	26.56	27.86

Supplementary Note 6. **Average exciton number calculation**

Measurement for the PL spectrum of individual sg-QDs were conducted using continuous-wave (cw) laser. The average exciton number with cw excition, $<N>_{cw}$, were calculated by following equation[11].

$$< N >_{cw} = \frac{ln2 \times P \times \tau_{average} \times \sigma_{abs}}{E_{photo} \times \pi r^2} \quad (3)$$

Where P is the power of laser, $\tau_{average}$ is the average exciton lifetime, σ_{abs} is the absorption cross section of QDs (3.29 x 10^{-14} for CdSe-ZnSe sg-QDs with r = 2.5 nm and H = 5 nm), E_{photon} is the energy of laser's photon (3.06 eV for 405 nm laser beam) and r is the radius of laser beam.

At the low excitation power, $\tau_{average} \fallingdotseq \tau_X$ (single exciton lifetime), but at the high excitation power, $\tau_{average}$ becomes shorter because of the multi excitons whose population is dependent for Poisson distribution. Therefore, the $\tau_{average}$ can be calculated by following equation.

$$\tau_{average} = \frac{\sum_n P(X=n) \times \frac{\tau_X}{n^2}}{\sum_k P(X=n)} \quad (4)$$

where P (X = n) is the probability of generation of n number of excitons following Poisson distribution.

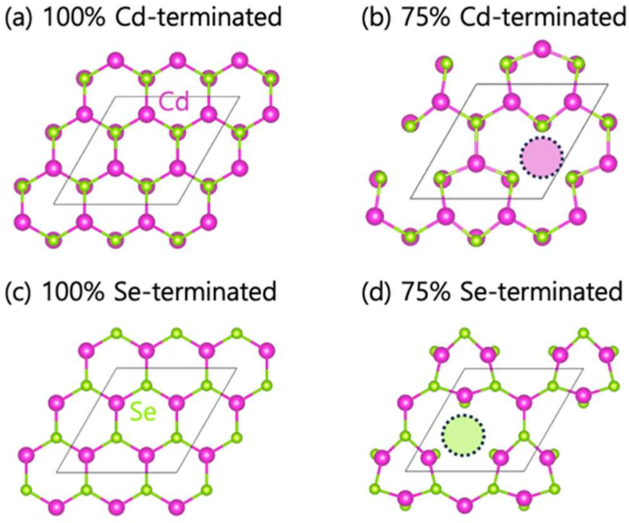

Supplementary Fig. 1 | Atomic structure of CdSe (111) surfaces: In (a) and (c), surfaces are 100 % Cd and Se-terminated, respectively. In (b) and (d), a Cd and a Se vacancy for every four surface atoms are generated, respectively. The sites of the vacancies are denoted by a dotted circle. Solid lines represent the cell boundary. Only the atoms at the surface are shown for clarity.

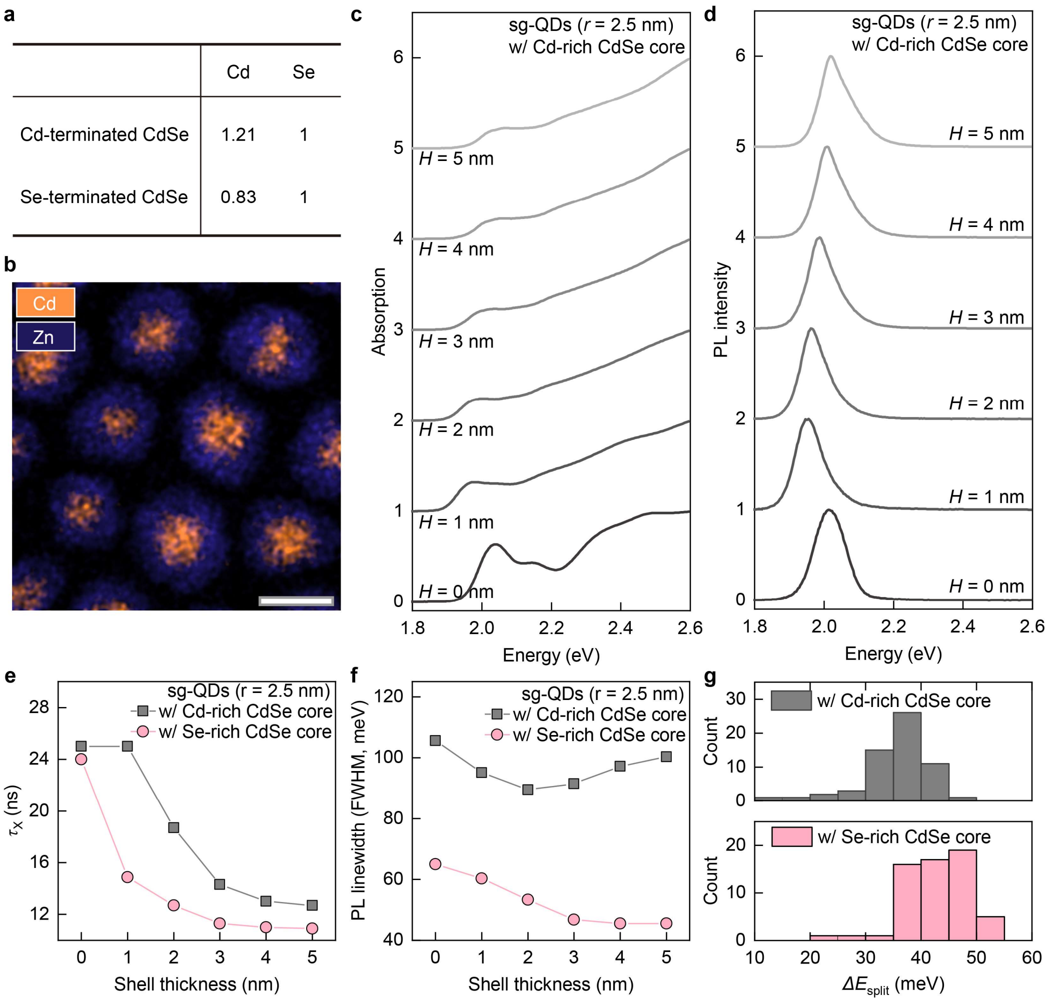

Supplementary Fig. 2 | (a) Inductively coupled plasma atomic emission spectroscopy (ICP-AES) elemental analysis showing the chemical compositions of Cd and Se in Cd-terminated CdSe and Se-terminated CdSe of the same radius (*r*) of 2.5 nm. (b) EDS elemental mapping for Cd-terminated CdSe-ZnSe sg-QDs. Scale bar is 10 nm. (c) Absorption, (d) PL spectra, (e) radiative single exciton recombination times (τ_X) and (f) PL linewidth of ensemble CdSe ($r = 2.5$ nm)-ZnSe sg-QDs with Cd-rich CdSe core upon ZnSe growth ($0 \leq H \leq 5.0$ nm). Optical characteristics of CdSe-ZnSe sg-QDs with Se-rich CdSe core ($r = 2.5$ nm) upon ZnSe shell growth (pink circles) are shown for comparison. (g) Heavy hole-light hole energy split (ΔE_{split}) measured from single-dot PL spectrum of 60 individual CdSe ($r = 2.5$ nm)-ZnSe ($H = 5.0$ nm) QDs with Cd-rich CdSe core (the mean split = 35.7 meV, upper panel) *versus* Se-rich CdSe core (the mean split = 42.8 meV, lower panel) are shown.

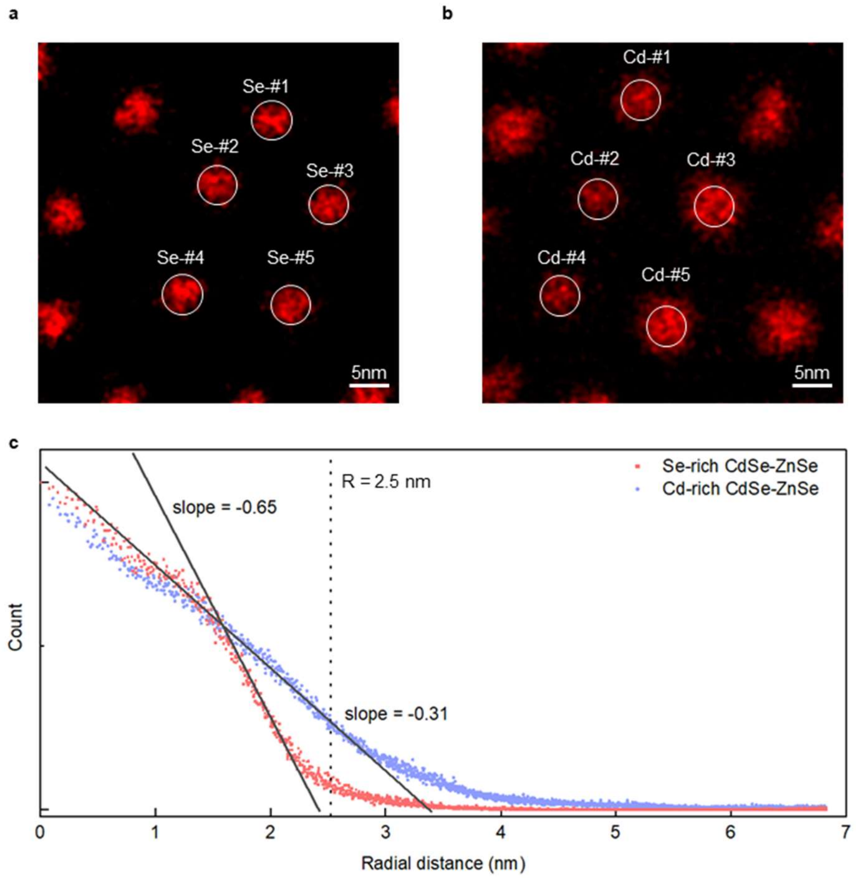

Supplementary Fig. 3 | EDS elemental mapping of CdSe (r = 2.5 nm)-ZnSe (H = 5.0 nm) core-shell sg-QDs grown from (a) CdSe core having Se rich surface *versus* (b) CdSe core having Cd rich surface. (c) Averaged radial distribution of Cd atom for 5 individual QDs extracted from (a) (red) and (b) (blue). Black lines represent linear fits to the curvature. Their slopes are noted in the figure.

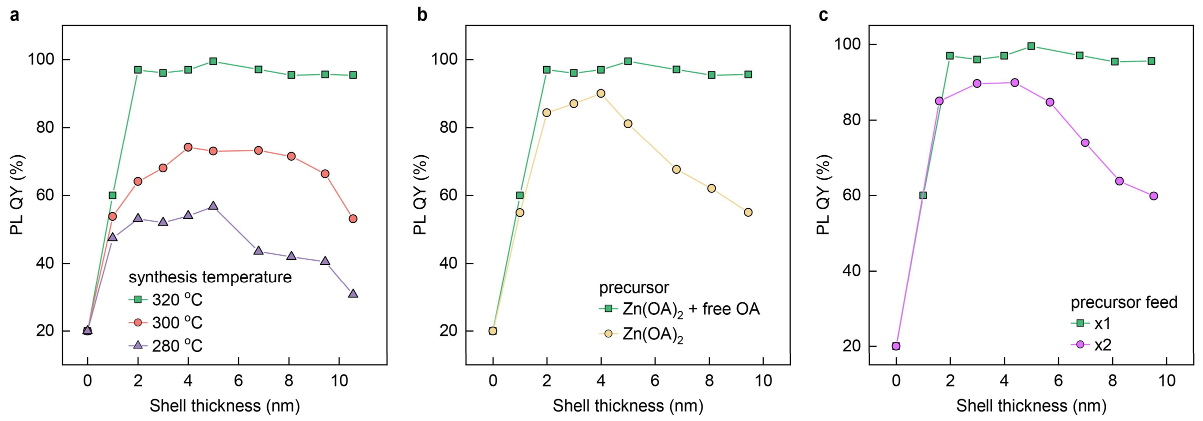

Supplementary Fig. 4 | PL QYs of CdSe-ZnSe QDs grown under different ZnSe growth conditions: (a) Reaction temperatures, (b) Zn precursor stoichiometry and (c) Zn and Se precursor feed rates. The optimal reaction condition (green square) is detailed in Methods, and other data sets are gained after varying the noted reaction parameter from the optimum condition.

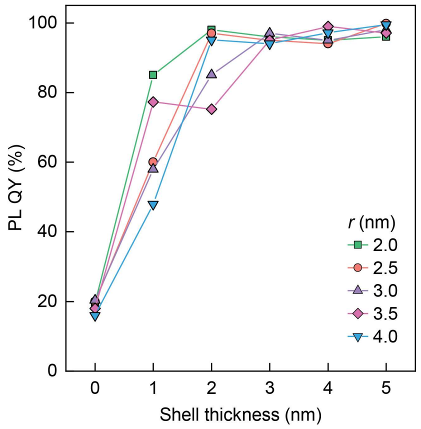

Supplementary Fig. 5 | Shell thickness dependent PL QYs of CdSe (2.0 nm ≤ r ≤ 4.0 nm)-ZnSe sg-QDs.

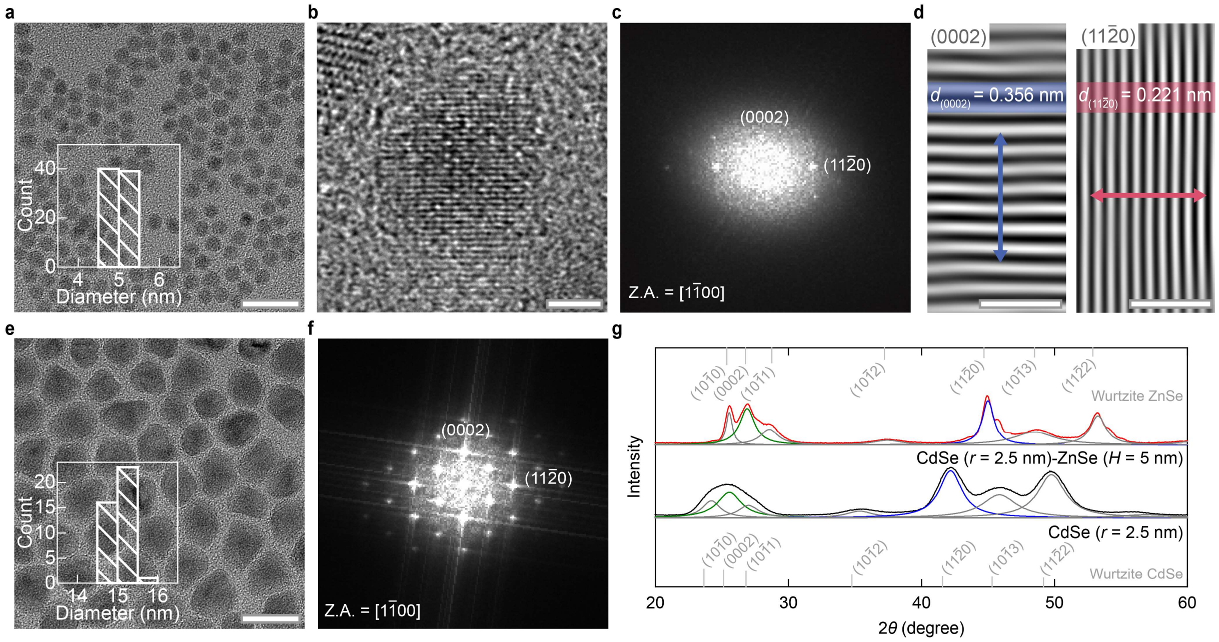

Supplementary Fig. 6 | (a,b) HR-TEM images, (c) FFT image and (d) inverse FFT images of CdSe core (r = 2.5 nm). Mean d-spacings along [0002] and [11$\bar{2}$0] are highlighted in (d). (e) HR-TEM image and (f) FFT image of CdSe (r = 2.5 nm)-ZnSe (H = 5.0 nm) sg-QDs shown in **Fig. 1c**. Scale bars in (a, b, d, e) indicate 20 nm, 2 nm, 1 nm and 20 nm, respectively. The insets in (a, e) show the size distribution of each QD sample. Zone axis of (c, f) are [1$\bar{1}$00]. (g) X-ray diffraction (XRD) patterns of wz-CdSe (r = 2.5 nm) cores and wz-CdSe (r = 2.5 nm)-wz-ZnSe (H = 5.0 nm) sg-QDs. XRD patterns of bulk wurtzite CdSe (bottom) and ZnSe (top) are shown for comparison.

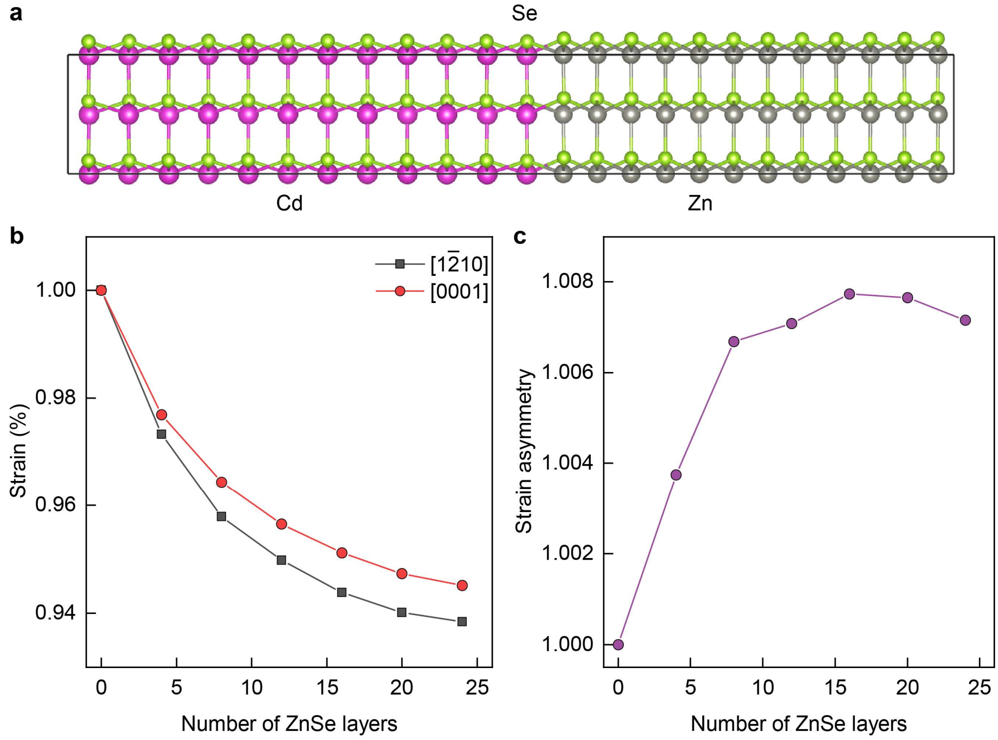

Supplementary Fig. 7 | (a) An atomistic model of CdSe/ZnSe interface, (b) The degree of biaxial strain along [0001] and [1$\bar{2}$10] directions, (c) Strain anisotropy, which is calculated by the ratio between the strain along the two directions.

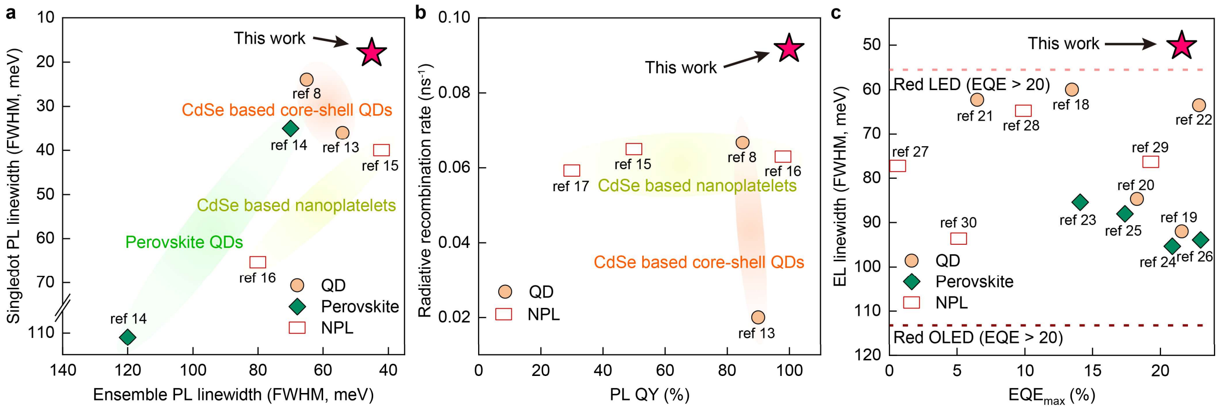

Supplementary Fig. 8 | (a) Spectral linewidths (single-dot *versus* ensemble levels) and (b) PL QYs and radiative recombination rates of colloidal nano-emitters: sg-QD in this works (star), CdSe-CdS QD[12], CdSe-CdZnSe QD[13] (circle), perovskite core QD[14], perovskite core-shell QD[14] (rhombus) and CdSe nanoplatelet[15] (NPL), CdSe-CdZnS NPL[16], CdSe-ZnS NPL[17] (rectangle). (c) EL linewidth versus maximum value of external quantum efficiency (EQE): sg-QD in this works (star), QD (circle)[18-22], perovskite (rhombus)[23-26] and NPL (rectangle)[27-30]. EL linewidths of red LED and OLED are 55 meV and 113 meV, respectively.

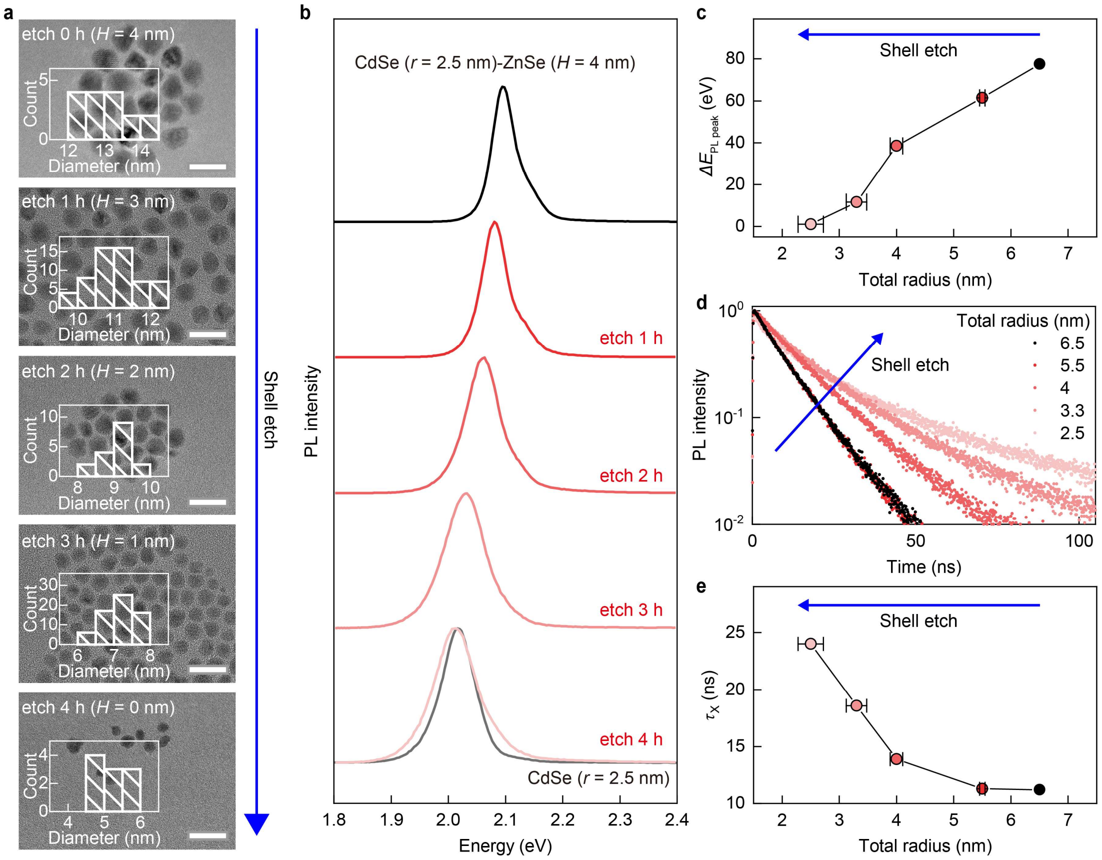

Supplementary Fig. 9 | (a) TEM images, (b) PL spectrum, (c) changes in the peak PL energy, (d) PL decay dynamics and (e) radiative recombination time upon chemical etching of CdSe (r = 2.5 nm)-ZnSe (H = 4.0 nm) QDs. Scale bars in (a) are 20 nm. It is noted that upon the shell etching, the peak PL energy, decay dynamics and exciton lifetimes return to the characteristics of original CdSe core-only QDs (r = 2.5 nm). The complete recovery of photophysical characteristics upon ZnSe shell etching indicate that the blue shift in PL and the acceleration of exciton lifetime seen in CdSe-ZnSe sg-QDs upon ZnSe growth are indeed attributed to the compressive strain imposed on CdSe core by ZnSe shell, rather than the irreversible compositional change in the core, for example, the inter-diffusion of Zn into CdSe to form CdZnSe core. Error bars in (c) and (e) are standard deviations.

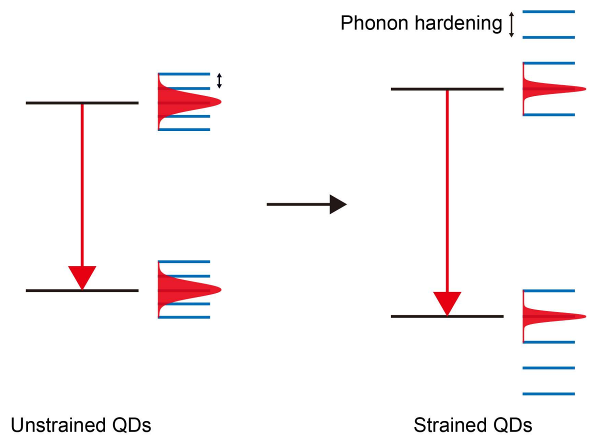

Supplementary Fig. 10 | Illustration depicting the mechanism of the narrowing of spectral linewidth in strained quantum dots (QDs), achieved through suppressed exciton-phonon coupling and increased optical phonon energies.

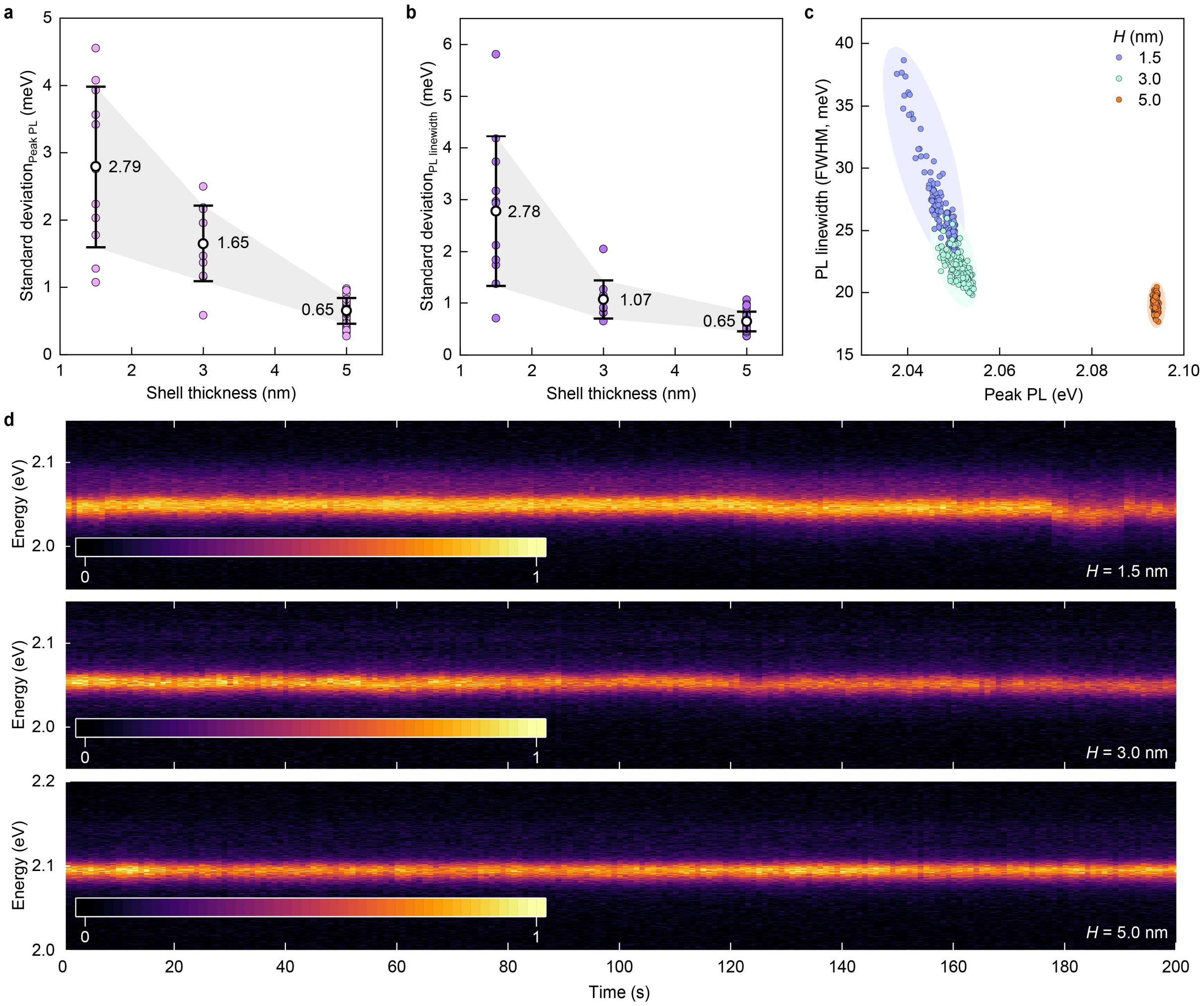

Supplementary Fig. 11 | Standard deviation of (a) peak PL and (b) PL linewidth in individual CdSe (r = 2.5 nm)-ZnSe sg-QDs extracted from PL spectra of 200 sequential frames (1s per each frame) according to shell thickness. The number of individual QDs are 11, 11 and 30, respectively. The mean values of calculated standard deviation are noted in the figure. In (a) and (b), symbols are averages, error bars are standard deviations, and shaded regions are connecting the error bars. (c) Peak PL *versus* PL linewidth and (d) 2D contour plot showing PL spectra of 200 sequential frames (1 s per each frame) for CdSe (r = 2.5 nm)-ZnSe (H, nm) sg-QDs with H = 1.5, 3.0 and 5.0 nm (from the top for (d)). The data in (c) are obtained from PL in (d). Shaded regions in (c) are guidance of range. The reduced standard deviation of peak PL and PL linewidth in relation to shell thickness suggests that Auger ionization, which can lead to the charging of QDs, is suppressed. In (c), there are red shifts accompanied by PL linewidth broadening for H = 1.5 and 3.0 nm which are associated with photocharging of QDs. In addition, the presence of thick energy barrier decreases the influence of Stark effect, which can change the distribution of electrons and holes, resulting in an altered spectrum.

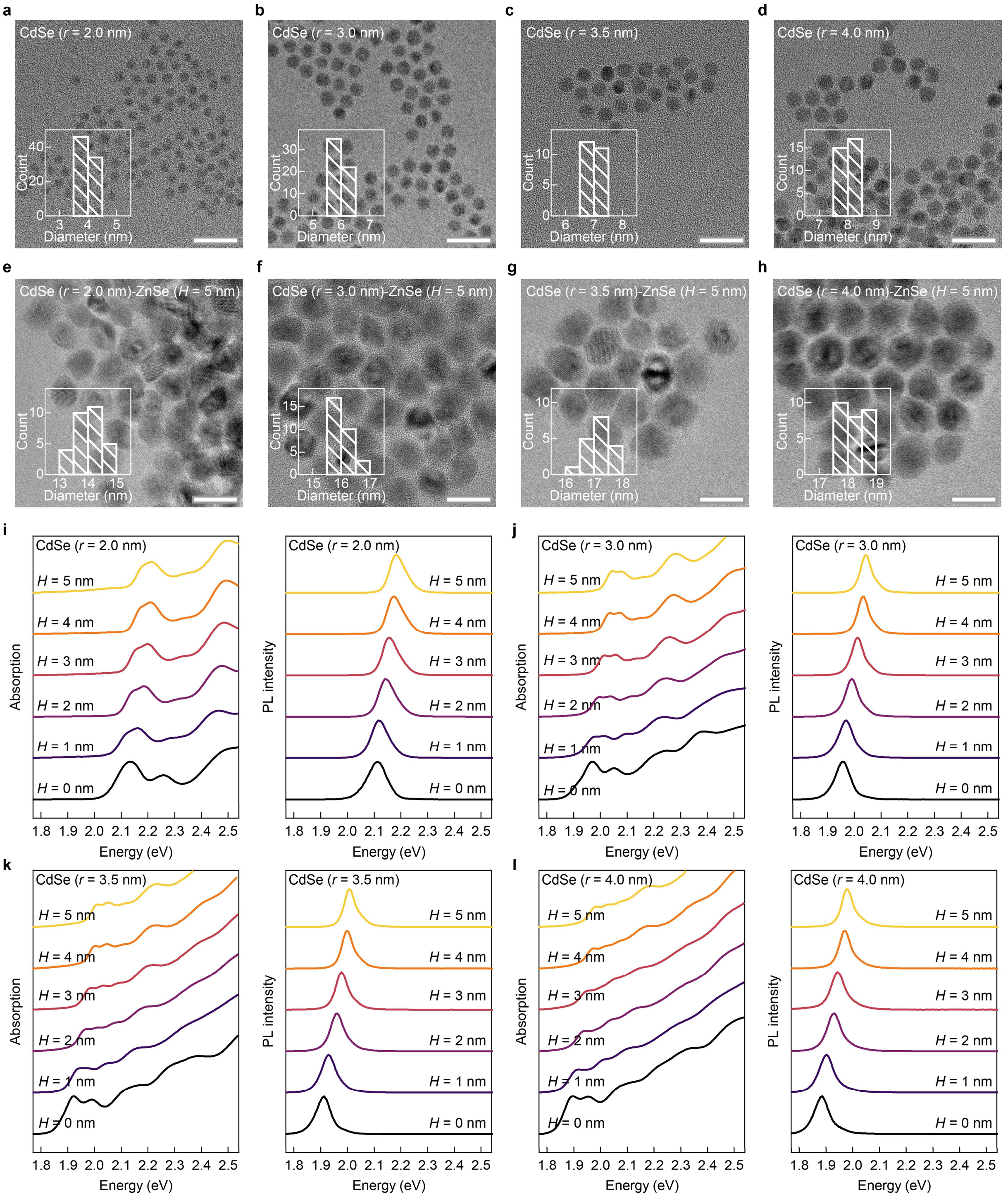

Supplementary Fig. 12 | HR-TEM images of (a-d) CdSe cores and (e-h) CdSe-ZnSe (H = 5.0 nm) sg-QDs with different core radii (r = (a,e) 2.0 nm, (b,f) 3.0 nm, (c,g) 3.5 nm and (d,h) 4.0 nm). The insets in (a-h) show size distribution of each particle. Scale bars in (a-h) are 20 nm. (i-l) Absorbance and PL spectra of CdSe-ZnSe sg-QDs upon ZnSe growth ($0 \leq H \leq 5.0$ nm).

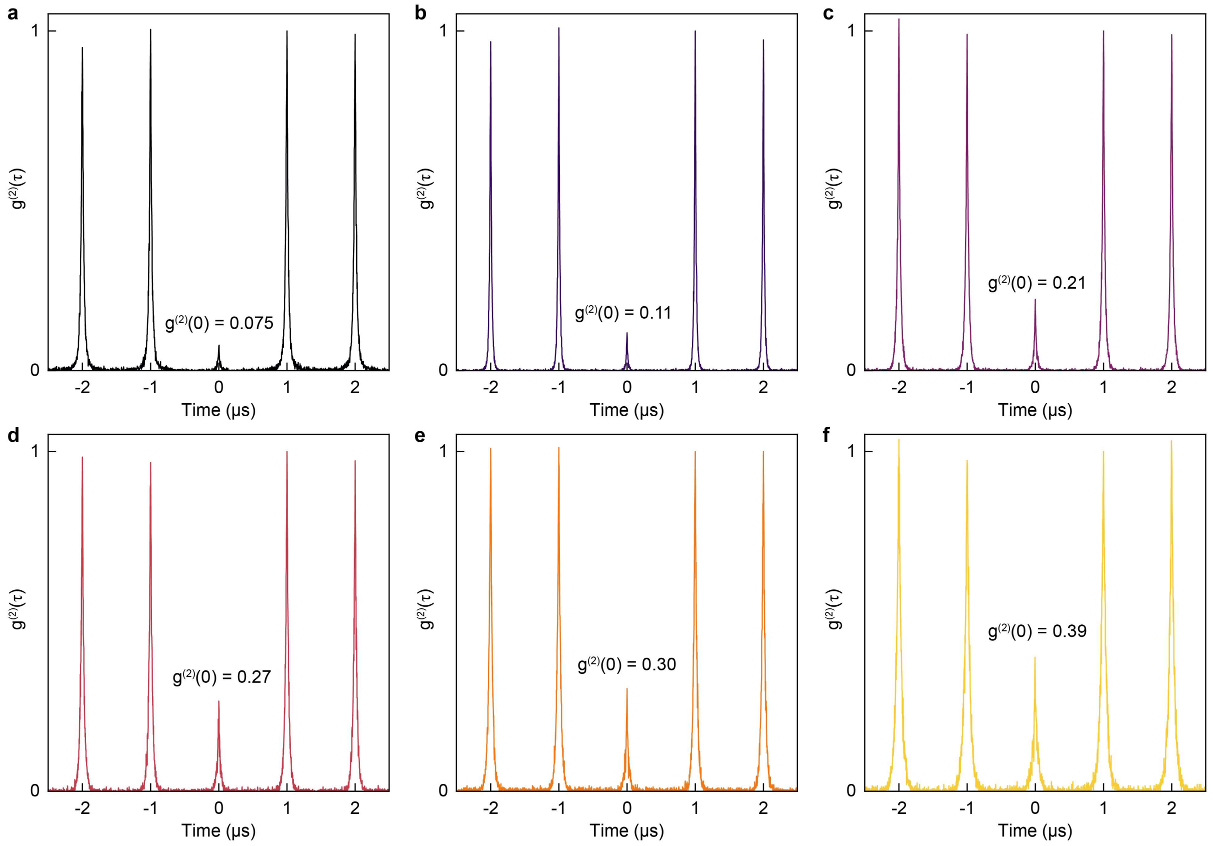

Supplementary Fig. 13 | The second-order correlation function graph of CdSe-ZnSe (*H* = 5.0 nm) sg-QDs with different core radii *r* = (a) 1.5 nm, (b) 2 nm, (c) 3 nm, (d) 3.5 nm, (e) 4 nm and (f) 4.5 nm. The values of $g^{(2)}(0)$ are noted in the figure.

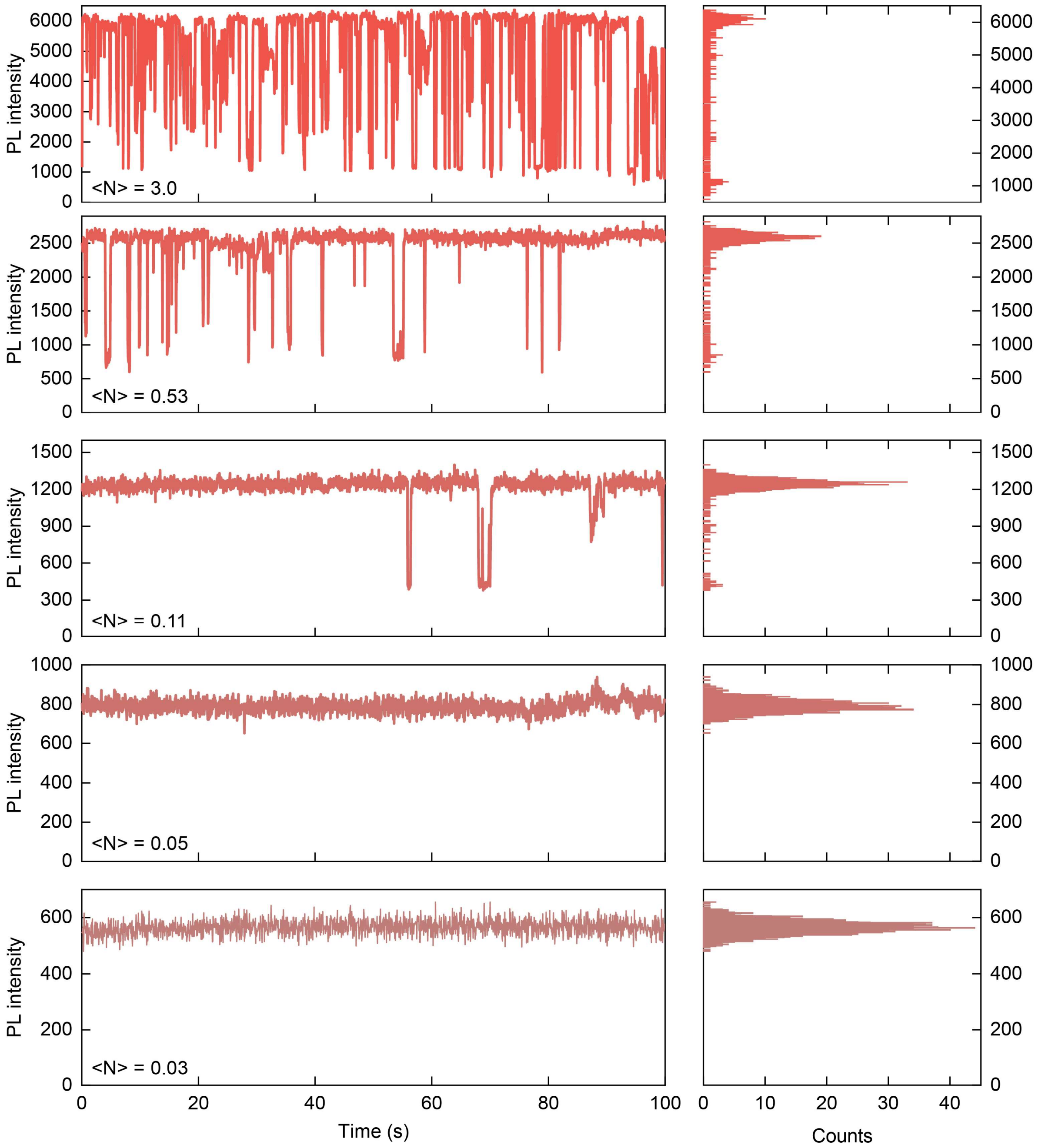

Supplementary Fig. 14 | Single-dot PL intensity time traces (left panel) and corresponding histogram (right panel) for CdSe (r = 2.5 nm)-ZnSe (H = 5.0 nm) sg-QD with increasing pump power of laser, which simultaneously increase <N>. The on-time fraction exceeds 80 for all pump powers.

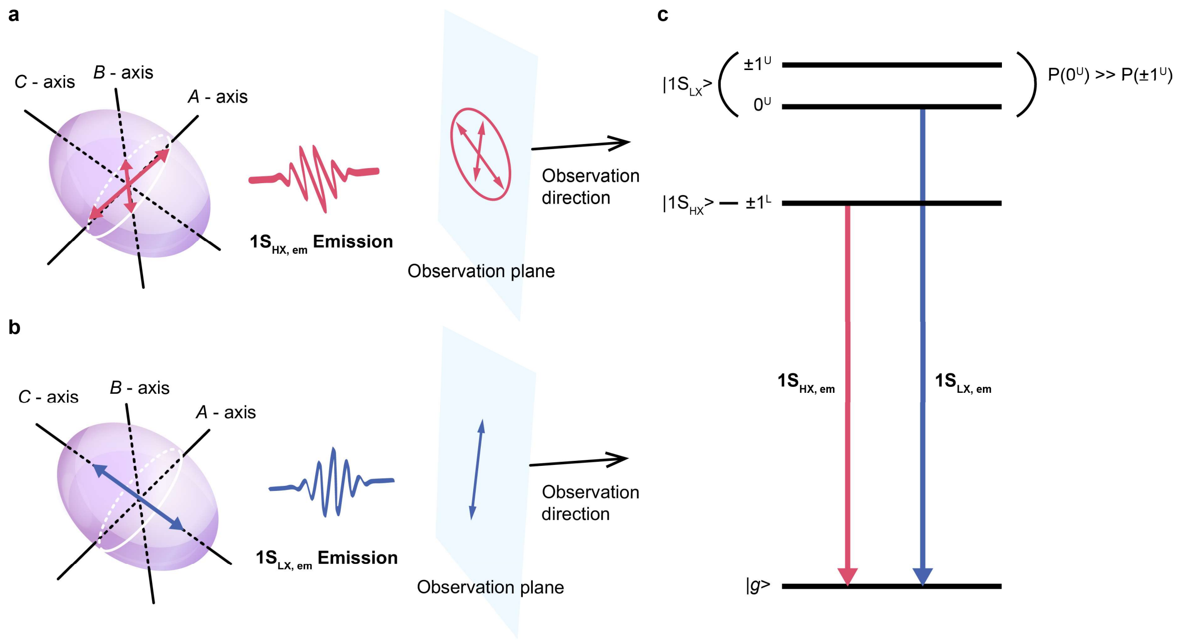

Supplementary Fig. 15 | Schematic representations of the transition dipole moments in the sg-QD and the polarization of their emission with projection onto the observation plane. (a) $1S_{HX,em}$ emission from 2D dipole on the *AB* plane of wurtzite crystal and (b) $1S_{LX,em}$ emission from 1D dipole along the *C* axis of wurtzite crystal. (c) The exciton fine structure of wurtzite CdSe with prolate spheroid shape[31]. For simplicity, only bright states are denoted. $|1S_{HX}>$ is composed of $\pm1^L$ which exhibit 2D dipole on the *AB* plane, while $|1S_{LX}>$ is composed with 0^U, which demonstrates 1D dipole along the *C* axis, and $\pm1^U$, which exhibit 2D dipole on the *AB* plane. Because the oscillator strength of 0^U is higher than that of $\pm1^U$, the emission from the $|1S_{LX}>$ is primarily dominated by 0^U component, supporting the dipole direction along the *C* axis for $1S_{LX,em}$.

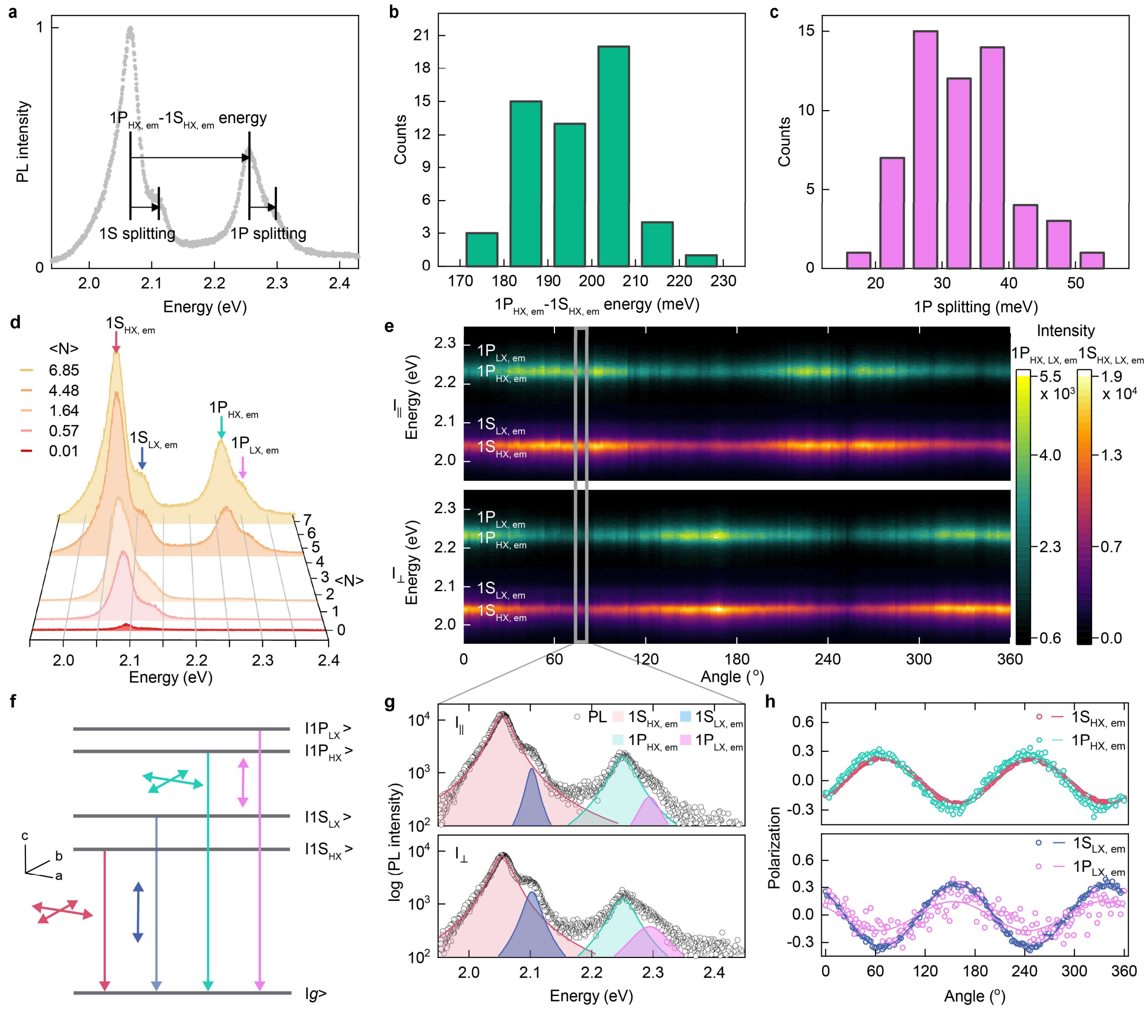

Supplementary Fig. 16 | (a) PL spectrum of a single CdSe (r = 2.5 nm)-ZnSe (H = 5.0 nm) sg-QD at $\langle N\rangle_{cw}$ = 6.85. 1S splitting, $1P_{HX,em} - 1S_{HX,em}$ energy and 1P splitting are indicated. (b) $1P_{HX,em} - 1S_{HX,em}$ energy histogram and (c) 1P splitting histogram of 57 individual CdSe (r = 2.5 nm)-ZnSe (H = 5.0 nm) sg-QDs. The average of $1P_{HX,em} - 1S_{HX,em}$ energy is 196 meV and the average of 1P splitting is 33.0 meV. (d) PL spectra of an individual sg-QD with varying exciton number densities ($\langle N\rangle_{cw}$ from 0.01 to 6.85). (e) 2D contour plots showing PL spectra ($I_{\parallel}$ and $I_{\perp}$ from the top) of the individual sg-QD ($\langle N\rangle_{cw}$ = 5.58) at varying rotation angles from 0 to 360 degree (100 ms per each degree). The spectra range is divided into 1S (1.92 - 2.15 eV) and 1P (2.15 - 2.41 eV) regions that are displayed with different scales and colors for visual clarity. $1S_{HX,em}$, $1S_{LX,em}$, $1P_{HX,em}$, and $1P_{LX,em}$ are indicated in each panel. (f) Energy level diagram of the optically active 1S ($|1S_{HX}\rangle$ and $|1S_{LX}\rangle$) and 1P ($|1P_{HX}\rangle$ and $|1P_{LX}\rangle$) excitonic states in the sg-QD. (g) PL spectra of $I_{\parallel}$ (upper panel) and $I_{\perp}$ (lower panel) at 80 degrees. Each spectrum is fitted with 4-Lorentzian curves to decouple $1S_{HX,em}$ (red line with shading), $1S_{LX,em}$ (blue line with shading), $1P_{HX,em}$ (green line with shading) and $1P_{LX,em}$ (pink line with shading). (h) DOP of $1S_{HX,em}$ (red open circle) and $1P_{HX,em}$ (green open circle) (upper panel) and $1S_{LX,em}$ (blue open circle) and $1P_{LX,em}$ (pink open circle) (lower panel). Each DOP is fitted with sine functions (solid lines in corresponding colors).

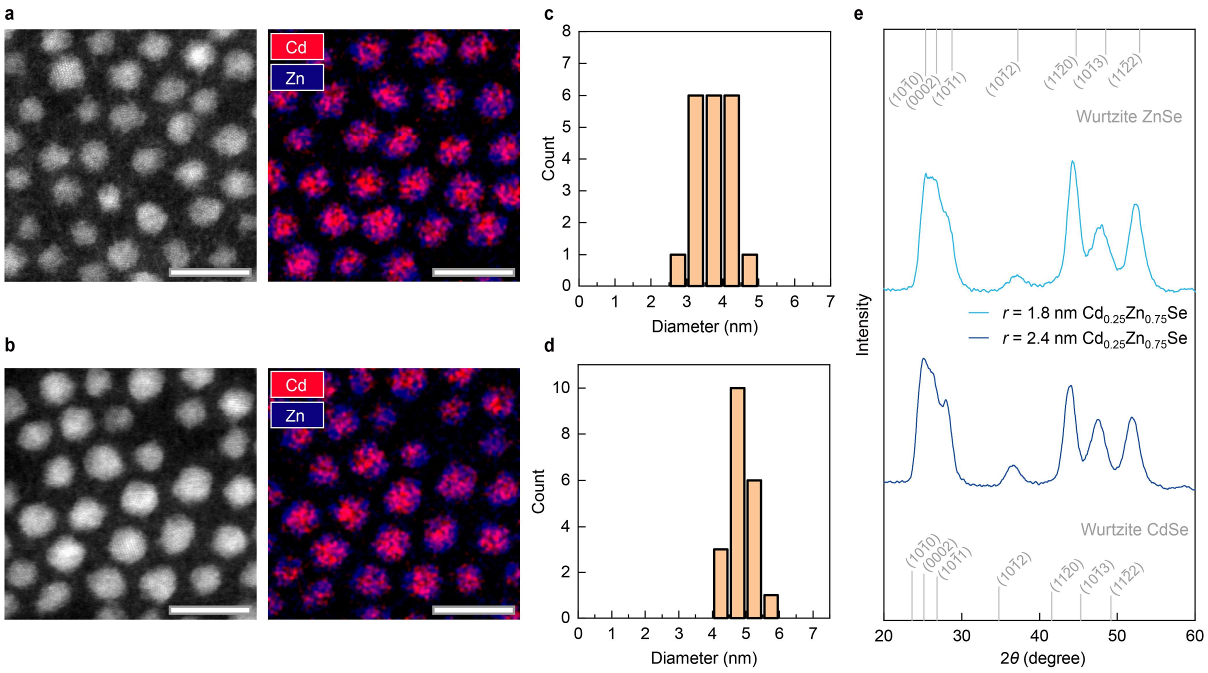

Supplementary Fig. 17 | TEM image (left panel), EDS elemental mapping (right panel) for $Cd_{0.25}Zn_{0.75}Se$ core with (a) r = 1.8 nm and (b) r = 2.4 nm. Scale bars in (a, b) are 10 nm. (c, d) Histogram of core diameter in (a, b), respectively. (e) XRD patterns of $Cd_{0.25}Zn_{0.75}Se$ core with r = 1.8 nm (sky blue) and r = 2.4 nm (deep blue) representing wurtzite crystal structures. Characteristics peaks for bulk CdSe and ZnSe are noted at the bottom and top.

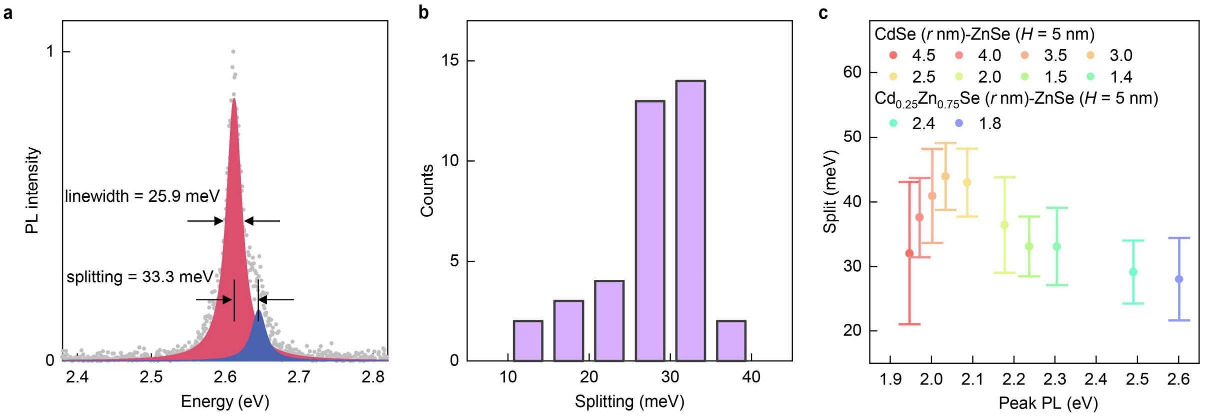

Supplementary Fig. 18 | (a) PL emission spectrum (grey dot) and its component, $1S_{HX,em}$ (red shading) and $1S_{LX,em}$ (blue shading), of a single $Cd_{0.25}Zn_{0.75}Se$ (r = 1.8 nm)-ZnSe (H = 5.0 nm) sg-QD. Emission linewidth for PL spectrum and the energy splitting are noted. (b) Histogram showing the energy split ($1S_{HX,em}$ -$1S_{LX,em}$) gained from 38 individual $Cd_{0.25}Zn_{0.75}Se$ (r = 1.8 nm)-ZnSe (H = 5.0 nm) sg-QDs. The mean split energy is *ca.* 28.0 meV. (c) $1S_{HX,em}$ -$1S_{LX,em}$ energy splits for the CdSe (1.4 nm ≤ r ≤ 4.5 nm)-ZnSe (H = 5.0 nm) sg-QDs and $Cd_{0.25}Zn_{0.75}Se$ (1.8 nm ≤ r ≤ 2.4 nm)-ZnSe (H = 5.0 nm) sg-QDs. Error bars are standard deviations.

Supplementary Table 3 | Comparison of photophysical characteristics of strain-graded CdSe-ZnSe QDs and compositionally-graded CdSe-$Cd_XZn_{1-X}Se$ QDs[8]

Key characteristics	Strain-graded QDs (this study)	Compositionally-graded QDs (ref. 8)
**Structure**	CdSe-ZnSe QDs with an abrupt interface	CdSe-$Cd_XZn_{1-X}Se$ QDs with composition gradient
**PL QY**	up to ~ 100 %	up to ~85 %
**Ensemble PL linewidth**	45 meV	68.6 meV
**Single-dot PL linewidth**	19.1 meV (in average)	24.1 meV (in average)
**Radiative decay rate**	0.092 ns^{-1}	0.063 ns^{-1}
**Heavy hole-light hole splitting**	42.8 meV	32.3 meV
**Single-dot PL on-time fraction**	95.80 %	~ 95 %
**Biexciton quantum yield***	7.3 % – 37.7 % (depending on the core radius)	~34 % (one example)
**Spectral diffusion (standard deviation of PL peak energy)**	0.65 meV	0.97 meV
**PL Peak**	460 nm - 640 nm	515 nm - 616 nm

*Biexciton quantum yields are calculated from following equation; $QY_{XX} = QY_X \times g^2(0)$.

Supplementary references

1 Blöchl, P. E. Projector augmented-wave method. *Physical Review B* **50**, 17953-17979 (1994).

2 Kresse, G. & Furthmüller, J. Efficient iterative schemes for ab initio total-energy calculations using a plane-wave basis set. *Physical Review B* **54**, 11169-11186 (1996).

3 Perdew, J. P., Burke, K. & Ernzerhof, M. Generalized Gradient Approximation Made Simple. *Physical Review Letters* **77**, 3865-3868 (1996).

4 Matthews, J. W. & Blakeslee, A. E. Defects in epitaxial multilayers: I. Misfit dislocations. *Journal of Crystal Growth* **27**, 118-125 (1974).

5 Osipiyan, Y. A. & Smirnova, I. Perfect dislocations in the wurtzite lattice. *physica status solidi (b)* **30**, 19-29 (1968).

6 Zakharov, O., Rubio, A., Blase, X., Cohen, M. L. & Louie, S. G. Quasiparticle band structures of six II-VI compounds: ZnS, ZnSe, ZnTe, CdS, CdSe, and CdTe. *Physical Review B* **50**, 10780-10787 (1994).

7 Adachi, S. *Handbook on physical properties of semiconductors*. (Springer Science & Business Media, 2004).

8 Heyd, J., Scuseria, G. E. & Ernzerhof, M. Hybrid functionals based on a screened Coulomb potential. *The Journal of Chemical Physics* **118**, 8207-8215 (2003).

9 Shan, W. *et al.* Pressure dependence of the fundamental band-gap energy of CdSe. *Applied Physics Letters* **84**, 67-69 (2003).

10 Togo, A., Chaput, L., Tadano, T. & Tanaka, I. Implementation strategies in phonopy and phono3py. *Journal of Physics: Condensed Matter* **35**, 353001 (2023).

11 Smyder, J. A. *et al.* The influence of continuous vs. pulsed laser excitation on single quantum dot photophysics. *Physical Chemistry Chemical Physics* **16**, 25723-25728 (2014).

12 Fan, F. *et al.* Continuous-wave lasing in colloidal quantum dot solids enabled by facet-selective epitaxy. *Nature* **544**, 75-79 (2017).

13 Park, Y. S., Lim, J. & Klimov, V. I. Asymmetrically strained quantum dots with non-fluctuating single-dot emission spectra and subthermal room-temperature linewidths. *Nat. Mater.* **18**, 249-255 (2019).

14 Rainò, G. *et al.* Ultra-narrow room-temperature emission from single CsPbBr3 perovskite quantum dots. *Nature Communications* **13**, 2587 (2022).

15 Tessier, M. D., Javaux, C., Maksimovic, I., Loriette, V. & Dubertret, B. Spectroscopy of Single CdSe Nanoplatelets. *ACS Nano* **6**, 6751-6758 (2012).

16 Tessier, M. D. *et al.* Spectroscopy of Colloidal Semiconductor Core/Shell Nanoplatelets with High Quantum Yield. *Nano Letters* **13**, 3321-3328 (2013).

17 Polovitsyn, A. *et al.* Synthesis of Air-Stable CdSe/ZnS Core–Shell Nanoplatelets with Tunable Emission Wavelength. *Chemistry of Materials* **29**, 5671-5680 (2017).

18 Lim, J., Park, Y.-S., Wu, K., Yun, H. J. & Klimov, V. I. Droop-free colloidal quantum dot light-emitting diodes. *Nano letters* **18**, 6645-6653 (2018).

19 Shen, H. *et al.* Visible quantum dot light-emitting diodes with simultaneous high brightness and efficiency. *Nature Photonics* **13**, 192-197 (2019).

20 Rhee, S. *et al.* Tailoring the electronic landscape of quantum dot light-emitting diodes for high brightness and stable operation. *Acs Nano* **14**, 17496-17504 (2020).

21 Rhee, S. *et al.* Steering interface dipoles for bright and efficient all-inorganic quantum dot based light-emitting diodes. *ACS nano* **15**, 20332-20340 (2021).

22 Liu, X. *et al.* Ultrastable and High-Efficiency Deep Red QLEDs through Giant Continuously Graded Colloidal Quantum Dots with Shell Engineering. *Nano Letters* **23**, 6689-6697 (2023).

23 Chiba, T. *et al.* Anion-exchange red perovskite quantum dots with ammonium iodine salts for highly efficient light-emitting devices. *Nature Photonics* **12**, 681-687 (2018).

24 Fang, Z. *et al.* Dual passivation of perovskite defects for light-emitting diodes with external quantum efficiency exceeding 20%. *Advanced Functional Materials* **30**, 1909754 (2020).

25 Lu, P. *et al.* ZnO–Ti3C2 MXene Electron Transport Layer for High External Quantum Efficiency Perovskite Nanocrystal Light-Emitting Diodes. *Advanced Science* **7**, 2001562 (2020).

26 Wang, Y. K. *et al.* All-inorganic quantum-dot LEDs based on a phase-stabilized α-CsPbI3 perovskite. *Angewandte chemie international edition* **60**, 16164-16170 (2021).

27 Chen, Z., Nadal, B., Mahler, B., Aubin, H. & Dubertret, B. Quasi-2D colloidal semiconductor nanoplatelets for narrow electroluminescence. *Advanced Functional Materials* **24**, 295-302 (2014).

28 Kelestemur, Y. *et al.* Colloidal CdSe quantum wells with graded shell composition for low-threshold amplified spontaneous emission and highly efficient electroluminescence. *ACS nano* **13**, 13899-13909 (2019).

29 Liu, B. *et al.* Record High External Quantum Efficiency of 19.2% Achieved in Light-Emitting Diodes of Colloidal Quantum Wells Enabled by Hot-Injection Shell Growth. *Advanced Materials* **32**, 1905824 (2020).

30 Qu, J. *et al.* Nanoplatelet-based light-emitting diode and its use in all-nanocrystal LiFi-like communication. *ACS applied materials & interfaces* **12**, 22058-22065 (2020).

31 Efros, A. L. *et al.* Band-edge exciton in quantum dots of semiconductors with a degenerate valence band: Dark and bright exciton states. *Physical Review B* **54**, 4843-4856 (1996).
